# In Vitro and In Vivo Effect of Peptides Derived from 14-3-3 *Paracoccidioides* spp. Protein

**DOI:** 10.3390/jof7010052

**Published:** 2021-01-13

**Authors:** Liliana Scorzoni, Ana Carolina Alves de Paula e Silva, Haroldo Cesar de Oliveira, Claudia Tavares dos Santos, Junya de Lacorte Singulani, Patricia Akemi Assato, Caroline Maria Marcos, Lariane Teodoro Oliveira, Nathália Ferreira Fregonezi, Diego Conrado Pereira Rossi, Leandro Buffoni Roque da Silva, Carlos Pelleschi Taborda, Ana Marisa Fusco-Almeida, Maria José Soares Mendes-Giannini

**Affiliations:** 1 School of Pharmaceutical Sciences, São Paulo State University (UNESP), Araraquara, São Paulo 14800-903, Brazil; liliscorzoni@yahoo.com.br (L.S.); ana_alpasi@hotmail.com (A.C.A.d.P.eS.); haroldocdoliveira@gmail.com (H.C.d.O.); claudiats@fcfar.unesp.br (C.T.d.S.); junyadelacorte@yahoo.com.br (J.d.L.S.); patricia.assato@gmail.com (P.A.A.); marcos_caroline@yahoo.com.br (C.M.M.); lariane.t@hotmail.com (L.T.O.); nat-fregonezi@hotmail.com (N.F.F.); ana.marisa@uol.com.br (A.M.F.-A.); 2 Department of Microbiology, Institute of Biomedical Sciences, University of São Paulo, São Paulo 05508-000, Brazil; rossido@ucmail.uc.edu (D.C.P.R.); leandro_br87@hotmail.com (L.B.R.d.S.); taborda@usp.br (C.P.T.)

**Keywords:** paracoccidioidomycosis, *Paracoccidioides* spp., 14-3-3 protein, *Galleria mellonella*, *Caenorhabditis elegans*, vaccine

## Abstract

Background: Paracoccidioidomycosis (PCM) is a chronic disease that causes sequelae and requires prolonged treatment; therefore, new therapeutic approaches are necessary. In view of this, three peptides from *Paracoccidioides brasiliensis* 14-3-3 protein were selected based on its immunogenicity and therapeutic potential. Methods: The in vitro antifungal activity and cytotoxicity of the 14-3-3 peptides were evaluated. The influence of the peptides in immunological and survival aspects was evaluated in vivo, using *Galleria mellonella* and the expression of antimicrobial peptide genes in *Caenorhabditis elegans*. Results: None of the peptides were toxic to HaCaT (skin keratinocyte), MRC-5 (lung fibroblast), and A549 (pneumocyte) cell lines, and only P1 exhibited antifungal activity against *Paracoccidioides* spp. The peptides could induce an immune response in *G. mellonella*. Moreover, the peptides caused a delay in the death of *Paracoccidioides* spp. infected larvae. Regarding *C. elegans*, the three peptides were able to increase the expression of the antimicrobial peptides. These peptides had essential effects on different aspects of *Paracoccidioides* spp. infection showing potential for a therapeutic vaccine. Future studies using mammalian methods are necessary to validate our findings.

## 1. Introduction

Globally, 1.2 million people are affected by fungal infections; however, it is difficult to estimate the real incidence of fungal infections since there is no obligatory reporting of these diseases in many countries [1,2]. Paracoccidioidomycosis (PCM) is a fungal disease restricted to Latin American countries, caused by the dimorphic fungi from the *Paracoccidioides* genus, composed of the species *Paracoccidioides brasiliensis, Paracoccidioides lutzii*, *Paracoccidioides americana*, *Paracoccidioides restrepiensis* and *Paracoccidioides venezuelensis* [3,4,5]. PCM is not a compulsory notification infection, which makes difficult the determination of real incidence of this disease. In Brazil it is estimated that the annual incidence ranges from 0.71 to 3.7/100,000 inhabitants [6,7].

The infection occurs by inhalation of fungus propagules and affects, in most cases, children/young adults (acute form) or adult men (chronic condition) [6]. This infection has important medical, social, and economic impact since it affects people during the productive life period and also can cause sequelae [8].

The clinical treatment for PCM includes itraconazole, cotrimoxazole (sulfamethoxazole/trimethoprim combination), and for severe cases, amphotericin B [7]. The treatment duration is long (9 to 18 months), and the cure is revealed by clinical, microbiological, radiological, and immunological criteria. Moreover, the patient should then undergo clinical and serological evaluation [7].

The adhesion process is the first step towards invasion and is fundamental to fungal infection success [9]. Adhesins are molecules or microbial surface structures that mediate the host-microorganism adhesion and, therefore, the interaction between these two organisms [10]. Even though *Paracoccidioides* spp. is not a mandatory intracellular pathogen, after adhesion, the fungus can be internalized, and this event is probably associated with the spread of the disease in different tissues [11].

Important adhesins have been described for *Paracoccidioides* spp. [12,13,14,15,16,17,18] and one of them is Pb 14-3-3, described as being involved in the adhesion to host cell and virulence process of *P. brasiliensis*. Aiming to demonstrate its role in the adhesion process, da Silva et al., [13] used the recombinant 14-3-3 protein. Its antibody showed adhesion inhibition and the localization of 14-3-3 protein in the fungal cell wall using in vitro and in vivo infection models. Assato et al. [17], using partial complementation of *Saccharomyces cerevisiae* with the *P. brasiliensis* 14-3-3 gene, demonstrated that the *P. brasiliensis* 14-3-3 protein behaved like an adhesin in this non-pathogenic yeast inducing *S. cerevisiae* adherence to epithelial cells. In addition, the *S. cerevisiae* strain showed increased expression of genes in the ergosterol pathway, the target for azoles [17]. Marcos et al. [18,19], using a 14-3-3- silenced strain of *P. brasiliensis*, demonstrated that in vitro 14-3-3 is important to the interaction with pneumocytes, and in vivo, showed less virulence in both *G. mellonella* and murine models, causing low granuloma and fungal burden in the last model. In this way, 14-3-3 protein is considered an important virulence factor of *Paracoccidioides* spp.

Due to ethical issues regarding the use of mammalian models in the research, invertebrate animal models have been well accepted by the scientific community. *G. mellonella* serves as an infection model for different human pathogenic fungi [20,21,22,23,24]. The *G. mellonella* immune system has a functional and structural similarity with mammalians [25], as the presence of cells denominated hemocytes with phagocytic ability. It produces melanin through the phenoloxidase enzyme, antimicrobial peptides, and superoxide as part of the humoral response [25,26,27,28]. *Caenorhabditis elegans* is a nematode susceptible to infection by pathogenic bacteria and fungi [29,30,31]. This nematode has been used to study virulence, filamentation, to search for new antifungal compounds, and to study their effectiveness [32,33,34]. Furthermore, *C. elegans* counts with an immune system that recognizes and eliminates pathogens and has high genetic homology with vertebrates [35]. The production of antimicrobial peptides such as *Ascaris suum* antibacterial factor (ASABF), caenacins (cnc), Neuropeptide-Like Protein (npl) class is an essential attribute of the *C. elegans* immune system in response to microorganisms [29,36,37,38].

The rising incidence of fungal infections caused by increasingly susceptible patients and different issues regarding actual available antifungal drugs (drug resistance, cost, toxicity, drug–drug interaction) [39,40,41,42] makes fungal prevention and treatment search urgent. There are vaccines against different microorganisms responsible for viral and bacterial diseases; however, there is no available vaccine against fungal infections. Based on this, this study aimed to determine candidates of immunogenic peptides from the 14-3-3 protein and evaluate its biological activity and immunogenic potential in vitro and in vivo.

## 2. Materials and Methods

### 2.1. Prediction of Binding Peptides from Paracoccidioides spp. 14-3-3 Protein to Major Histocompatibility Complex (MHC) Class II Mouse Alleles

For the in silico analysis, the NetMHCIIpan-4.0 server was used (https://services.healthtech.dtu.dk/service.php?NetMHCIIpan-4.0). The mouse alleles H-2-Iad, H-2-IAb, H-2-IAk, H-2-IAq, H-2-IAs and H-2-IAu were selected. These analyses resulted in three peptides that demonstrated the right binding prediction, here designated as P1, P2, and P3. The peptides were commercially purchased from Peptide 2.0 (Chantilly, VA, USA) for the characterization and biological activity evaluation.

### 2.2. Microorganisms and Culture Conditions

*Paracoccidioides brasiliensis* (São Paulo, Brazil) strain 18 and *P. lutzii* Pb01-like (American Type Culture Collection—ATCC MYA-826/Goiania, Brazil) strain 01 from Laboratory of Clinical Mycology, Faculty of Pharmaceutical Sciences, São Paulo State University (UNESP), Araraquara, Brazil were maintained in Fava-Netto agar at 37 °C for 4–5 days. For the experiments, the Fava-Netto agar culture was transferred to a brain heart infusion (BHI) broth supplemented with 1% glucose for 3–4 days at 37 °C at 150 rpm.

### 2.3. Cytotoxicity of 14-3-3 Peptides

Peptides cytotoxicity was performed using the resazurin method, according to Pavan et al. 2010 [43]. For this assay, HaCaT (skin keratinocyte), MRC-5 (lung fibroblast), and A549 (pneumocyte) cell lines, all acquired from the collection of the Banco de Células do Rio de Janeiro—BCRJ, Brazil, were used. For monolayer formation, 10^6^ cells/mL were plated in a 96-well microplate using Dulbecco’s Modified Eagle Medium (DMEM; Gibco; Thermo Fisher Scientific, Carlsbad, CA, USA) supplemented with 10% fetal bovine serum (FBS) and incubated for 24 h at 37 °C with 5% CO_2_. Next, the supernatant was removed, the peptides diluted in DMEM at concentrations from 19.5 to 2500 µg/mL were added. As controls were used DMEM medium, cells plus DMEM medium, and cells treated with 30% dimethyl sulfoxide (DMSO). The plate was incubated for 24 h at 37 °C with 5% CO_2_. After the incubation, 30 µL/well of 0.01% resazurin were added and incubated for six h at 37 °C with 5% CO_2_. The readings were taken at 570/600 nm, and the results expressed as a percentage of cell viability.

### 2.4. Antifungal Activity of 14-3-3 Peptides

The antifungal activity of the 14-3-3 peptides was evaluated by broth microdilution method, according to de Paula e Silva, et al. [44]. For this, Roswell Park Memorial Institute (RPMI) 1640 medium with l-glutamine and without sodium bicarbonate buffered at pH 7 with 4-Morpholinepropanesulfonic acid (MOPS) and supplemented with 2% glucose was used. The *Paracoccidioides* spp. inoculum concentration was 0.5–2.5 × 10^3^ cells/mL, and the antifungal activity of the peptides P1, P2 and P3 from 4.9 to 2500 µg/mL were evaluated. Amphotericin B from 0.016 to 8 µg/mL was used as a control. The plates were incubated at 37 °C with shaking at 150 rpm for 72 h. After that, 20 µL/well of Alamar Blue^TM^ (BioSource International, Invitrogen, Eugene, OR, USA) was added, followed by another 24 h incubation. The readings were taken at 570/600 nm, and the results expressed as a percentage of fungal cell viability.

### 2.5. Paracoccidioides spp. Adhesion Inhibition Assay to the Extracellular Matrix Component or Pneumocytes (A549) Cells

Then adhesion inhibition assay was performed according to De Oliveira et al. [45]. For this, 96-well Corning^®^ plates were coated with 50 µg/mL laminin (Sigma Aldrich, Saint Louis, MO, USA) for 18 h at 4 °C. Wells plates were washed three times with phosphate-buffered saline (PBS). Then, the laminin-coated plate was treated with 100 µg/mL of peptides P1, P2, and P3 for 1 h at 37 °C. *Paracoccidioides* spp. at 10^6^ cells/mL were stained with 100 µM of 5-(and-6)-carboxyfluorescein diacetate succinimidyl ester (CFDA-SE) (Sigma Aldrich, Saint Louis, MO, USA) for 20 min at 37 °C, then 100 µL of this inoculum was added to laminin-coated wells and treated with the peptides and incubated for 3 h at 37 °C. After incubation, the wells were washed carefully three times with PBS to remove fungal cells not adhered to the laminin. One hundred microliters of PBS were added to each well, and with the aid of a tip, the bottom of each well was scraped to remove the yeast adhered to the laminin, this process was repeated twice. The samples were analyzed by flow cytometry using BD FACSCanto equipment (BD Biosciences, San Jose, CA, USA) by acquiring 30 s in medium mode; afterward, the data were calculated for milliliters. For the evaluation of the effects of peptides in *Paracoccidioides* spp. adhesion to pneumocytes (A549) cells, 10^6^ cells/mL were plated in 96-well microplate using Modified Eagle Medium supplemented with 10% FBS and incubated for 24 h at 37 °C with 5% CO_2_. Treatments were realized as described above in the laminin assay. The samples were analyzed by flow cytometry and expressed in fungal–pneumocyte percentage of interaction.

### 2.6. G. mellonella Rearing and Experiment Conditions

*G. mellonella* larvae were cultivated in the laboratory, according to Jorjão, et al. [46]. Larvae weighting 150–200 mg, active and without dark spots, were selected and incubated at 37 °C, and protected from the light before the experiments. Before treatment/infection, aseptic treatment of the pro-legs was performed with 70% ethanol. The larvae were inoculated and/or treated using 10 µL Hamilton syringes (Hamilton, Reno, NV, USA). In all treatments, larvae were incubated at 37 °C. For each experimental condition, 10–15 larvae were used, and each experiment was repeated at least three times.

#### 2.6.1. Toxicity Evaluation of 14-3-3 Peptides

For toxicity, *G. mellonella* larvae were treated with peptides P1, P2, or P3 at concentrations 10, 20, 40, 100, and 200 µg/larva and incubated at 37 °C. Survival was evaluated daily for seven days, as described above.

#### 2.6.2. Hemocytes Concentration

*G. mellonella* were treated with 100 µg/larva of peptides P1, P2, and P3 and incubated for 3 h at 37 °C. After this period, the larvae hemolymph was removed through an incision with the aid of a scalpel, 10 µL of hemolymph was diluted in 90 µL of anticoagulant solution (2% NaCl; 0.1 M glucose; 30 mM sodium citrate; 26 mM citric acid and 10 mM Ethylenediamine tetraacetic acid (EDTA)) and then to 200 µL of FACs fluid. Hemocyte counts were performed by flow cytometry in BD FACSCanto equipment (BD Biosciences, San Jose, CA, USA). For that, the number of events was recorded in 60 s on medium acquisition mode, which is equivalent to 60 µL of the sample; afterward, the data were calculated for milliliters. The hemocytes from larvae injected with PBS were compared with the infected ones.

#### 2.6.3. Induction of Phenoloxidase Activity by Peptides

The quantification of the phenoloxidase enzyme was performed according to the protocol described by Laughton and Siva-Jothy [47]; for that, the larvae of *G. mellonella* were treated with 100 µg/larva for 3 h at 37 °C. Then the hemolymph of 5 larvae was collected and pooled; 50 µL of each hemolymph pool was added to 150 µL anticoagulant solution and centrifuged for 5 min at 500 rpm to remove the cells. Aliquots of 50 µL of the supernatant were collected, and 50 µL of PBS was added to the samples; for positive control, 50 µL LPS (lipopolysaccharide) at 2.5 mg/mL, was added instead of PBS, and the plate was incubated for 5 min at 25 °C. After incubation, 25 µL of 6 mM l-dopa was added as a substrate in all samples and incubated for an additional 1 h at 25 °C. The reading was performed at 490 nm, and the optical density values obtained were analyzed.

#### 2.6.4. Analysis of the Expression of Antimicrobial Peptides

Five *G. mellonella* larvae per group were treated with 100 µg/larva of peptides P1, P2, and P3 and incubated for 3 h at 37 °C. The hemolymph of all larvae was pooled and diluted in 100 µL of anticoagulant solution. RNA extraction was performed using the Trizol reagent (Invitrogen Life Technologies, Carlsbad, CA, USA). The synthesis of the cDNA strand was performed using reverse transcriptase (RevertAid H Minus Reverse Transcriptase, (Fermentas, Waltham, MA, USA). Real-time polymerase chain reaction (PCR) was performed with Maxima^®^ SYBR Green/ROX qPCR Master Mix (2X) (Fermentas, Waltham, MA, USA) using the Applied Biosystems 7500 equipment. The relative expression of the galiomicin and gallerimycin genes was calculated using values 2^−ΔΔCT^ according to Livak and Schmittgen [48], using s7 gene (ribosomal protein) as a housekeeping gene. The primers sequences are described in Table 1.

#### 2.6.5. Efficacy of 14-3-3 Peptides in *G. mellonella* Model

The larvae were treated with 100 µg/larva of peptides P1, P2, and P3 and incubated for 3 h at 37 °C. After that, the infection with 5 × 10^6^ cells/larvae of *P. brasiliensis* or *P. lutzii* was done, and as a control, larvae were treated with PBS. After the infection, larvae were incubated at 37 °C, and death was monitored daily for seven days, checking the movement after touching them with forceps.

### 2.7. Effect of 14-3-3 Peptides on the Induction of C. elegans Antimicrobial Peptides

*C. elegans* strain N2 (wild type) synchronized in stage L4 was treated with 250 µg/mL of each of the peptides. After 6 h of treatment, the worms were washed with 50 mM NaCl to remove the peptides. RNA extraction, cDNA synthesis, and real-time PCR were performed as described above. The relative expression of the *abf-1*, *abf-3 cnc-4*, *npl- 27,* and *npl-31* was calculated by 2^−ΔΔCT^ values according to Livak and Schmittgen [48], using actin (*act-1*) as housekeeping gene. The primers sequences are described in Table 2.

### 2.8. Statistical Analysis

Statistical analysis was performed using GraphPad Prism 6 software (GraphPad Software, Inc., La Jolla, CA, USA). The effects of peptides in hemocytes concentration and phenoloxidase liberation were analyzed by using Kruskal–Wallis and Dunn’s tests, and *C. elegans* antimicrobial peptides real-time PCR. The survival test was analyzed using the Log-rank test (Mantel–Cox). The *p* > 0.05 is considered significant in all tests.

## 3. Results

### 3.1. In Silico Prediction of Binding Peptides from Paracoccidioides spp. 14-3-3 Protein to Mouse MHC Class II Alleles

**Figure 1:**
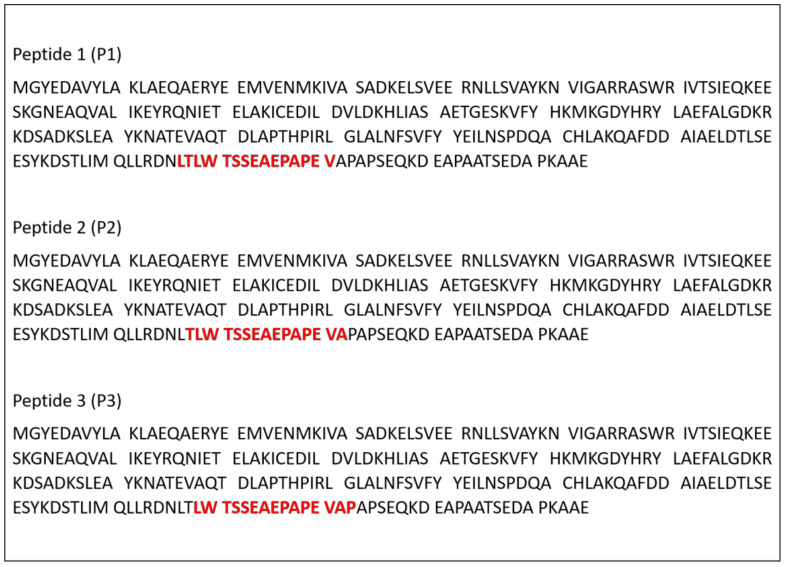
Immunogenic peptides of protein 14-3-3 (GenBank: AY462124.1, PADG_04056 - 14-3-3 family protein epsilon) selected by in silico analysis.

The NetMHCIIpan-4.0 algorithm revealed three peptides with a good prediction for binding to mouse MHC class II alleles. The sequences of these peptides are in red (Figure 1), and these were called peptide 1 (P1), peptide 2 (P2), peptide 3 (P3).

### 3.2. Effect of 14-3-3 Peptides in Mammalian Cell

**Figure 2:**
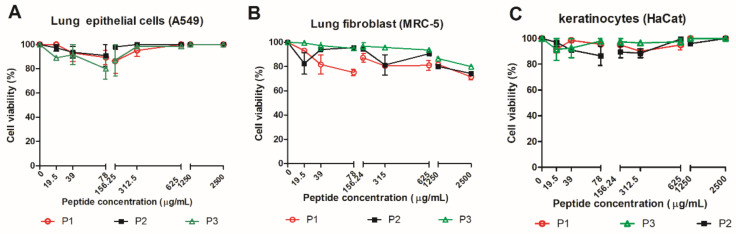
Cytotoxicity of 14-3-3 peptides evaluated in lung epithelial cells (**A**), lung fibroblasts (**B**), and skin keratinocytes (**C**), using the resazurin method. Data expressed as mean ± standard deviation of two biological replicates and two independent experiments.

PCM affects the lungs and skin during the infection; because of this, the peptides from 14-3-3 protein were evaluated regarding its cytotoxicity in lung epithelial cells, lung fibroblasts, and skin keratinocytes at concentrations ranging from 19.5 to 2500 µg/mL (Figure 2A–C). The peptides treatment showed viability rates above 80% for all evaluated cell lines, regardless of concentration.

### 3.3. Effect of 14-3-3 Peptides on Paracoccidioides spp. Cells

**Figure 3:**
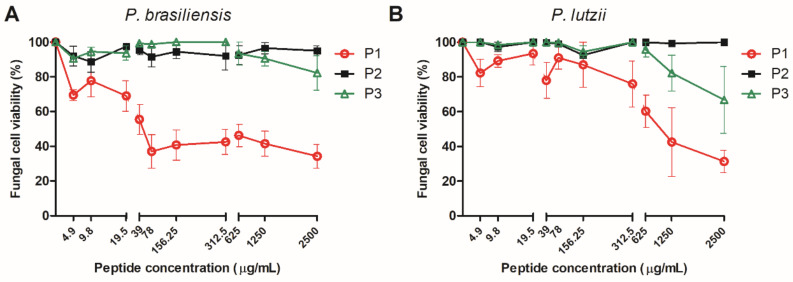
Growth inhibition of 14-3-3 protein peptides (P1, P2, P3) evaluated by microdilution assay against *P. brasiliensis* (**A**) and *P. lutzii* (**B**) concentrations ranging from 4.9 to 2500 µg/mL. Data expressed as mean ± standard deviation of two biological replicates and two independent experiments.

The broth microdilution assay demonstrated that peptide P1 was the most active peptide and reduced *P. brasiliensis* and *P. lutzii* viability in a concentration-dependent manner. The treatment with 4.9 µg/mL of P1 reduced the *P. brasiliensis* viability by 30.5% and 2500 µg/mL was able to reduce *P. brasiliensis* viability by 66%. In a similar way, P1 could reduce the *P. lutzii* viability by 22% (39 µg/mL) to 70% (2500 µg/mL) (Figure 3A,B). The peptide P2 did not affect *Paracoccidioides* spp. viability. On the other hand, P3 at high concentration (2500 µg/mL) reduced *P. brasiliensis* and *P. lutzii* viability by 20% and 30%, respectively.

### 3.4. Evaluation of the Immunomodulatory and Antifungal Effect in G. mellonella

Before the evaluation of immunomodulatory and antifungal effect in *G. mellonella*, the toxicity of the 14-3-3 peptides was assessed at concentrations of 10, 20, 40, 100 µg/larva. No larval death was observed in survival curves at the evaluated concentrations, demonstrating that these peptides were not toxic (data not shown). The peptides concentration of 100 µg/larva were selected for the next *G. mellonella* assays.

#### 3.4.1. Effect of 14-3-3 Peptides in Hemocytes Concentration

**Figure 4:**
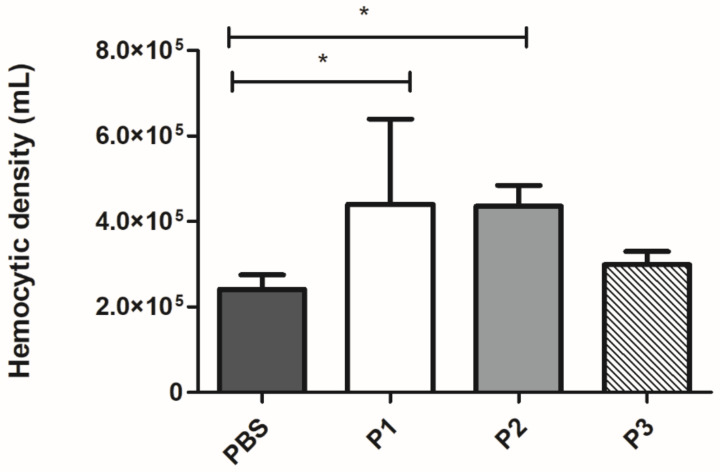
Effect of 14-3-3 peptides in hemocyte concentration of *G. mellonella* larvae. Data expressed as mean ± standard deviation of eight to 10 biological replicates and three independent experiments. * *p* < 0.05 using Kruskal–Wallis and Dunn’s test.

After 3 h of treatment with 100 µg/larvae of 14-3-3 protein peptides, a 1.8-fold increase in *G. mellonella* hemocytes concentration (*p* < 0.05) was observed for peptides P1 and P2 (Figure 4) when compared to PBS. No statistical difference was observed for the treatment with P3.

#### 3.4.2. Effect of 14-3-3 Protein Peptides in Humoral Response of *G. mellonella*

**Figure 5:**
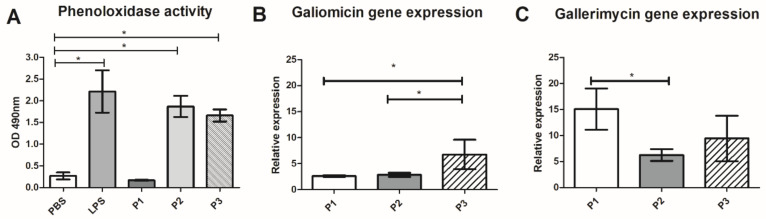
Effect of 14-3-3 protein peptides in humoral response of *G. mellonella.* Induction of *G. mellonella* phenoloxidase enzyme after treatment of 14-3-3 peptides using l-dopa as substrate (**A**). Relative expression *G. mellonella* of peptides galiomicin (**B**) and gallerimycin (**C**) genes after treatment of 14-3-3 peptides. Data expressed as mean ± standard deviation of three biological replicates and three independent experiments (* *p* < 0.05), using Kruskal-Wallis and Dunn’s for phenoloxidase enzyme activity and gene expression using Student’s *t*-test. Lipopolysaccharide (LPS).

As described, phenoloxidase enzyme and antimicrobial peptides are essential factors in the humoral response *of G. mellonella* against microorganisms. The effect of 14-3-3 peptides in the humoral response of *G. mellonella* was evaluated in phenoloxidase enzyme and in the expression of genes that encode the antimicrobial peptides galiomicin and gallerimycin. P2 and P3 induce the production of phenoloxidase with a 7.0 and 6.3-fold increase (*p* < 0.05), respectively when compared to larvae treated with PBS (Figure 5A). Also, P1, P2, and P3 increased galiomicin and gallerimycin genes expression (Figure 5B,C). The most significant increase in peptide expression occurred with the treatment of P1 and P3, respectively, a 15-fold increase for galerimicin and a 6.8-fold increase in galiomycin expression compared to the untreated control.

#### 3.4.3. Antifungal Protective Effect of 14-3-3 Protein Peptides against *Paracoccidioides* spp. Infection

**Figure 6:**
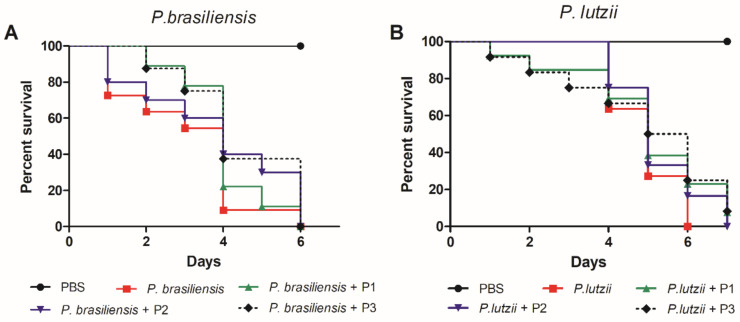
Survival curve of *G. mellonella* treated with immunogenic peptides at a concentration of 100 µg/larva at 3 h before the infection with *P. brasiliensis* (**A**) or *P. lutzii* (**B**). Log-rank test (Mantel–Cox) of eight to 10 biological replicates and three independent experiments (*p* < 0.05).

We also evaluated whether the changes in the immune response of *G. mellonella* caused by pretreatment with the peptides would affect the survival of larvae infected with *P. brasiliensis* and *P. lutzii*, which are the most studied etiologic agents of PCM. Although there may have been no statistical difference between the curves, all three peptides led to a delay in the larvae death (Figure 6).

### 3.5. Evaluation of C. elegans Antimicrobial Peptide Expression after Treatment with 14-3-3 Peptides

**Figure 7:**
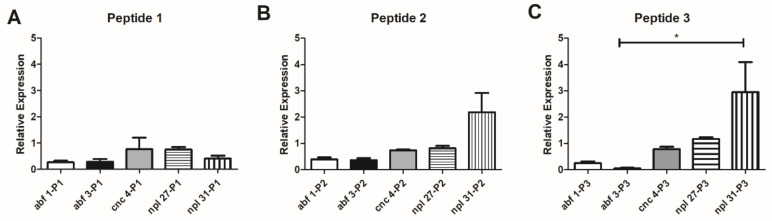
Relative expression of *abf-1*, *abf-3 cnc-4*, *npl- 27* and *npl-31* genes related to the production of *C. elegans* antimicrobial peptides when in contact with P1 (**A**), P2 **(B**) or P3 (**C**). Data are expressed as mean ± standard deviation of three biological replicates and three independent experiments (* *p* < 0.05) using Kruskal–Wallis and Dunn’s.

The effect of peptides 14-3-3 was evaluated in the *C. elegans* model, and peptide P3 induced a significant increase of *npl-31* (2.9 times), compared to other antimicrobial peptides (*p* < 0.05) (Figure 7).

Table 3 summarizes the results obtained in vitro and in *G. mellonella* and *C. elegans* invertebrate models.

## 4. Discussion and Conclusions

Mycoses are a threat to immunosuppressed and immunocompetent patients [2,54]. PCM is the most prominent systemic mycosis in Latin America due to its high incidence, long period of treatment, and the sequelae that can affect patients in the most productive life period [6,7,8]. Considering these factors, the search for alternatives to prevention and treatment is urgent. Different approaches have been investigated in the search for a safe and effective immunization or vaccination therapy for invasive mycoses such as the use of monoclonal antibodies [55], peptide vaccine [56,57], vaccines based on nanotechnology [58], and DNA vaccine [59].

The 14-3-3 protein is an important virulence factor of *Paracoccidioides* spp. acting as an essential adhesin [17,18,19]. Therefore, we aimed to determine candidates of immunogenic peptides from this protein and evaluate their biological activity and immunogenic potential in vitro and in vivo. Three peptides of 14-3-3 protein were obtained based on MHC class II alleles binding properties using in silico methods. The cytotoxicity was analyzed using skin keratinocytes, lung fibroblasts, and pneumocytes. The peptides were not toxic for these cells, leading to a small reduction (20%) viability. Therefore, a reduction in viability below 30% is acceptable in cytotoxicity testing, according to ISO 10993-5 (2009).

**Figure A1:**
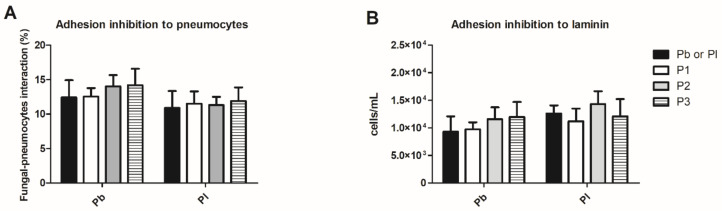
Effect of 14-3-3 peptides treatment in the adhesion to pneumocytes (**A**) or laminin (**B**). Data are expressed as mean ± standard deviation of eight biological replicates and three independent experiments using Kruskal–Wallis and Dunn’s (*p* < 0.05).

The antifungal activity against *Paracoccidioides* spp. showed that the peptide P1 was the most effective in reducing *Paracoccidioides* spp. viability around 30.5–70% at concentrations between 4.9 and 2500 µg/mL. On the other hand, the pneumocytes treatment with 14-3-3 peptides followed by *Paracoccidioides* spp. infection was not effective at inhibiting the host–cell interaction of *P. brasiliensis* or *P. lutzii*. Similar results were observed when the extracellular matrix component (laminin) was treated with 14-3-3 peptides and further infected (Figure A1). This peptide originates from an adhesin of the fungus itself, and possibly is not associated with the adhesion role. Thus, inhibition of adhesion to the components of the extracellular matrix or interaction with cells is not a property of this peptide.

Next, 14-3-3 peptides characterization was expanded for in vivo assay using the *G. mellonella* invertebrate model. The 14-3-3 peptides were not toxic for the larvae and could increase the larvae hemocyte density compared to the control. The increase in the hemocyte density is an essential aspect of the cellular immune response in *G. mellonella* associated with eliminating infections [60]. Previous data showed that infection by *Paracoccidioides* spp. reduces the density of hemocytes [22]; therefore, the induction of these cells is an essential strategy for treating larvae infected with this fungus. The challenge of *G. mellonella* with a sublethal dose of *Aspergillus fumigatus* increased the hemocyte concentration and the resistance of larvae to this infection [61]. Hemocyte concentration increases with the peptides’ treatment derived from a phage library with anti-adhesive activity for *Paracoccidioides* spp. [62]. The treatment of *G. mellonella* infected with *Candida haemulonii* with copper (II), manganese (II), and silver (I) 1,10-phenanthroline chelates significantly increased the hemocyte concentration, larvae survival, and reduced fungal burden [63]. Treatment and pretreatment of larvae infected with *Candida auris* with crude extract and derivatives of the culture supernatant fraction of *Lactobacillus paracasei* 28.4, a postbiotic, increased hemocytes level of infected larvae [64].

In our study, we demonstrated that P2 and P3 were able to induce phenoloxidase production. Melanization is a vital process in the immune response of invertebrates. It occurs by activating the pro-phenoloxidase cascade generating toxic intermediates such as quinones, and the final product is melanin. Various components of the membrane and cell wall of microorganisms such as PAMPS (pathogen associated molecular patterns), peptidoglycans, lipopolysaccharide, and β-1-3 glucan (cell wall lipopolysaccharides) participate in the activation of the pro-phenoloxidase. Also, enzymes released by the invertebrate itself due to tissue damage by microorganisms can stimulate the cascade [65]. These substances are very toxic to microorganisms and help in phagocytosis and invertebrate survival [66]. As already described, *G. mellonella* larvae infected with a sublethal dose of *C. albicans* could induce phenoloxidase activity [67].

Another essential attribute of the *G. mellonella* immune system is the production of antimicrobial peptides. In this study, 14-3-3 peptides increased the expression of gallerimycin and galiomicin peptides. A significant increase of galiomicin occurred after treatment with P1. The P3 peptide was able to increase the expression of both antimicrobial peptides. The relation between these antimicrobial peptides with infection control has already been demonstrated in different microorganisms. The pretreatment of *G. mellonella* larvae with *Photorhabdus luminescens* killed by heat with subsequent infection by this bacterium increased gallerimycin and galiomycin expression and, consequently, the survival of the larvae [68]. Another study demonstrated that sublethal doses of *C. albicans* and *A. fumigatus* induced the expression of the same antimicrobial peptides [50,61]. Furthermore, the infection of *G. mellonella* with *Bacillus subtilis* and *Bacillus atrophaeus* vegetative and sporulating forms also increased gallerimycin and galliomycin expression [69]. The pre-infection with *B. subtilis* and *B. atrophaeus* increased gallerimycin and galiomicin expression and survival of larvae infected with *C. albicans.* On the other hand, the treatment with peptides delayed larvae death when they were infected with *P. brasiliensis* or *P. lutzii*.

The effect of 14-3-3 peptides was also evaluated on the expression of *C. elegans* antimicrobial peptides. The treatment promoted an increase of peptides from *Ascaris suum* antibacterial factor (ASABF), caenacins (cnc), neuropeptide-Like protein (npl) class. Different signaling cascades are involved in the *C. elegans* response against pathogens, such as pathways involving protein kinases (MAPKs), homologous to TGF-β, insulin-like peptides, and Toll-like receptors. These cascades are responsible for the expression of antimicrobial peptides [52]. An increasing number of reports have demonstrated the importance of these peptides’ expression in protecting *C. elegans* against different fungal and bacterial pathogens [51,53,70,71]; thus, 14-3-3 peptides proved to be beneficial in *C. elegans*.

Due to the fungus’ complexity, attenuated, or inactivated, whole cells for immunization can generate unwanted responses. Predictions of epitope biology for selecting peptides capable of inducing the immune response are alternatives in searching for vaccines [57]. A significant advance in the research of a possible PCM vaccine was made using one peptide of the gp43 *P. brasiliensis* protein (P10), which is an adhesin and significant diagnostic antigen of this mycosis. By using different vaccine approaches, the P10 peptide was able to reduce the fungal burden and to increase cytokine production [59,72,73,74,75].

This study characterized three peptides derived from the protein 14-3-3 of *P. brasiliensis*. The peptides induced an immune response in *G. mellonella* and *C. elegans*, invertebrate animal models demonstrating their potential. Although a positive correlation has been reported between invertebrates and mammals’ models [30], further studies are needed to evaluate these peptides in this model.

## Figures and Tables

**Table 1:** Primers sequence of *G. mellonella* antimicrobial peptides.

Gene	Forward Primer	Reverse Primer	References
S7	ATG TGC CAA TGC CCA AGT TG	GTG GCT AGG CTT GGG AAG AAT	[49]
Galiomicin	TCG TAT CGT CAC CGC AAA ATG	GCC GCA ATG ACC ACC TTT ATA	[50]
Gallerimycin	TATCAT TGG CCT TCT TGG CTG	GCA CTCGTA AAA TAC ACA TCC GG	[50]

**Table 2:** Primers sequence of *C. elegans* antimicrobial peptides.

Gene	Forward Primer	Reverse Primer	Reference
*abf-1*	GTACAGCACAGAAATGCATGACCGG	GGCGTTTGAACAACCTCCACAGAAGC	[51]
*abf-3*	GGTGTCGAATAAGGCAGTGTGGACCT	GGCATTTCCATAGCTATCCCTGTAGC	[51]
*cnc-4*	ACAATGGGGCTACGGTCCATAT	ACTTTCCAATGAGCATTCCGAGGA	[52]
*nlp-27*	CGGTGGAATGCCATATGGTG	ATCGAATTTACTTTCCCCATCC	[53]
*nlp-31*	GGTGGATATGGAAGAGGTTATGGAG	GTCTATGCTTTTACTTTCCCC	[53]
*act-1*	CCATCATGAAGTGCGACATTG	CATGGTTGATGGGGCAAGAG	[53]

**Table 3:** Summary of the 14-3-3 peptides effect in vitro and invertebrate models.

Peptide	Mammalian Cell Toxicity	*G. mellonella* Toxicity	Antifungal Activity	Hemocytes Density	Phenoloxidase Activity	Expression *G. mellonella* Antimicrobial Peptides	Protective effect against *Paracoccidioides* spp. infection in *G. mellonella* model	Expression of Antimicrobial Peptides of *C. elegans*
P1	Non-toxic (A549, HACat and MRC-5)	Non-toxic (10–100 µg/larva)	*P. brasiliensis* (30.5–66% of viability reduction) *P. lutzii* (22–70% of viability reduction)	1.8-fold increase	No statistical difference	Galiomicin: 2.6-fold-increase Gallerimycin:15-fold-increase	Delay in the larvae death	*abf-1:* 0.26-fold-increase *abf-3*: 0.29-fold- increase *cnc-4*: 0.77-fold- increase *npl-27*: 0.75-fold- increase *npl-31*: 0.40-fold increase
P2	Non-toxic (A549, HACat and MRC-5)	Non-toxic (10–100 µg/larva)	No antifungal activity against *Paracoccidioides* spp.	1.8-fold increase	7-fold increase	Galiomicin: 2.8 fold-increase Gallerimycin: 6.2 fold-increase	Delay in the larvae death	*abf-1:* 0.39-fold-increase *abf-3*: 0.36-fold- increase *cnc-4*: 0.73-fold- increase *npl-27*: 0.81-fold- increase *npl-31*: 2.17-fold increase
P3	Non-toxic (A549, HACat and MRC-5)	Non-toxic (10–100 µg/larva)	*P. brasiliensis* (20% of viability reduction) *P. lutzii* (30% of viability reduction at 2500 µg/mL)	No statistical difference	6.3-fold increase	Galiomicin: 6.7 folds-increase Gallerimycin: 6.8-fold increase	Delay in the larvae death	*abf-1:* 0.20-fold-increase *abf-3*: 0.05-fold- increase *cnc-4*: 0.78-fold- increase *npl-27*: 1.16-fold- increase *npl-31*: 2.9-fold increase

## Data Availability

The data presented in this study are available in the article.
